# Effects of prone positioning on lung mechanical power components in patients with acute respiratory distress syndrome: a physiologic study

**DOI:** 10.1186/s13054-024-04867-6

**Published:** 2024-03-15

**Authors:** Christoph Boesing, Joerg Krebs, Alice Marguerite Conrad, Matthias Otto, Grietje Beck, Manfred Thiel, Patricia R. M. Rocco, Thomas Luecke, Laura Schaefer

**Affiliations:** 1 Department of Anesthesiology and Critical Care Medicine, University Medical Center Mannheim, Medical Faculty Mannheim of the University of Heidelberg, Theodor-Kutzer-Ufer 1-3, 68167 Mannheim, Germany; 2 Laboratory of Pulmonary Investigation, Carlos Chagas Filho Institute of Biophysics, Federal University of Rio de Janeiro, Centro de Ciências da Saúde, Avenida Carlos Chagas Filho, 373, Bloco G-014, Ilha Do Fundão, Rio de Janeiro, Brazil

**Keywords:** Acute respiratory distress syndrome, Respiratory mechanics, Prone positioning, Ventilator-induced lung injury, Lung-protective ventilation, Mechanical power, End-expiratory lung volume

## Abstract

**Background:**

Prone positioning (PP) homogenizes ventilation distribution and may limit ventilator-induced lung injury (VILI) in patients with moderate to severe acute respiratory distress syndrome (ARDS). The static and dynamic components of ventilation that may cause VILI have been aggregated in mechanical power, considered a unifying driver of VILI. PP may affect mechanical power components differently due to changes in respiratory mechanics; however, the effects of PP on lung mechanical power components are unclear. This study aimed to compare the following parameters during supine positioning (SP) and PP: lung total elastic power and its components (elastic static power and elastic dynamic power) and these variables normalized to end-expiratory lung volume (EELV).

**Methods:**

This prospective physiologic study included 55 patients with moderate to severe ARDS. Lung total elastic power and its static and dynamic components were compared during SP and PP using an esophageal pressure-guided ventilation strategy. In SP, the esophageal pressure-guided ventilation strategy was further compared with an oxygenation-guided ventilation strategy defined as baseline SP. The primary endpoint was the effect of PP on lung total elastic power non-normalized and normalized to EELV. Secondary endpoints were the effects of PP and ventilation strategies on lung elastic static and dynamic power components non-normalized and normalized to EELV, respiratory mechanics, gas exchange, and hemodynamic parameters.

**Results:**

Lung total elastic power (median [interquartile range]) was lower during PP compared with SP (6.7 [4.9–10.6] versus 11.0 [6.6–14.8] J/min; *P* < 0.001) non-normalized and normalized to EELV (3.2 [2.1–5.0] versus 5.3 [3.3–7.5] J/min/L; *P* < 0.001). Comparing PP with SP, transpulmonary pressures and EELV did not significantly differ despite lower positive end-expiratory pressure and plateau airway pressure, thereby reducing non-normalized and normalized lung elastic static power in PP. PP improved gas exchange, cardiac output, and increased oxygen delivery compared with SP.

**Conclusions:**

In patients with moderate to severe ARDS, PP reduced lung total elastic and elastic static power compared with SP regardless of EELV normalization because comparable transpulmonary pressures and EELV were achieved at lower airway pressures. This resulted in improved gas exchange, hemodynamics, and oxygen delivery.

*Trial registration*: German Clinical Trials Register (DRKS00017449). Registered June 27, 2019. https://drks.de/search/en/trial/DRKS00017449

**Supplementary Information:**

The online version contains supplementary material available at 10.1186/s13054-024-04867-6.

## Background

The improved survival associated with prone positioning (PP) in patients with moderate to severe acute respiratory distress syndrome (ARDS) in the PROSEVA trial [1] has been attributed to a reduction in overdistension and cyclical airway opening and closing [2–4]. Combining PP and protective ventilation with positive end-expiratory pressure (PEEP) to improve ventilation distribution [5] may therefore limit ventilator-induced lung injury (VILI) [2, 3, 6]. PP reduces the pleural pressure gradient and decreases the dependence on PEEP to homogenize lung ventilation [7] by increasing transpulmonary pressures (*P*_TP_) and end-expiratory lung volume (EELV) [8]. This may result in reduced mechanical power (MP), defined as the mechanical energy delivered by the ventilator to the respiratory system [9, 10], and MP components normalized to EELV [11]. MP integrates static (respiratory system peak, plateau, and driving pressures [Δ*P*_RS_], PEEP, and tidal volume) and dynamic (airflow amplitude, inspiratory time fraction, and respiratory rate) components and has been considered a unifying driver of VILI [9, 10].

PP and supine positioning (SP) may affect static and dynamic MP components differently due to changes in pleural pressures and lung and chest wall mechanics [7, 12–14]; however, to date, no study has evaluated whether these changes have an impact on the transmission of lung MP components and their normalization to EELV, which reflects the energy transfer per aerated lung volume.

To clarify this issue, the current study compared the following parameters during PP and SP: lung total elastic power and its components (elastic static power and elastic dynamic power) and these variables normalized to EELV when using an esophageal pressure (*P*_eso_)-guided ventilation strategy [5]. We hypothesized that PP combined with protective ventilation reduces lung total elastic power and its static and dynamic components, as well as the energy transfer per aerated lung volume in patients with moderate to severe ARDS. The primary endpoint was the effect of PP on lung total elastic power non-normalized and normalized to EELV. Secondary endpoints were the effects of PP and different ventilation strategies in SP on elastic static and dynamic power components non-normalized and normalized to EELV, respiratory mechanics, EELV, gas exchange, and hemodynamic parameters.

## Methods

This prospective, physiologic study was conducted as part of a multipurpose study [8], with independent research questions and study protocol, approved by the local ethical committee (Medizinische Ethikkomission II, University Medical Center Mannheim, Medical Faculty Mannheim of the University of Heidelberg, Mannheim, Germany; registration number 2018-609N-MA-Amend3) and after study registration at the German Clinical Trials Register (DRKS00017449, https://drks.de/search/en/trial/DRKS00017449).

### Patients

Consecutive patients with moderate to severe ARDS (defined by the ratio of arterial partial pressure of oxygen to the fraction of inspired oxygen [PaO_2_/FiO_2_] ≤ 150 mmHg according to the Berlin definition [15]) were enrolled between July 2019 and June 2023. Informed consent was obtained from the legal representative of each patient before enrollment. Exclusion criteria were age < 18 years, pregnancy, end-stage chronic organ failure, inherited cardiac malformations, severe head injury, and severe hemodynamic instability.

### Clinical management

All patients were sedated with sufentanil (20–30 μg/h) and midazolam (10–20 mg/h) to achieve a score of − 5 on the Richmond Agitation-Sedation Scale, and complete neuromuscular blockade was maintained throughout the study period with cisatracurium. An esophageal balloon catheter (NutriVent nasogastric catheter; Sidam, Mirandola, Italy) was advanced into the stomach, inflated, and withdrawn into the esophagus until the appearance of cardiac artifacts on the pressure tracing [16]. The balloon was inflated with the lowest volume to obtain the largest swings in *P*_eso_ during tidal ventilation. *P*_eso_ measurements were considered reliable if the ratio of change in *P*_eso_ to change in airway pressure was 0.8–1.2 during an end-expiratory occlusion test [17, 18].

A thermodilution catheter (5F Pulsiocath, Pulsion Medical Systems, Munich, Germany) was inserted via the femoral artery in all patients included in the study to allow hemodynamic measurements with a pulse contour cardiac output monitor (PiCCOplus; Pulsion Medical Systems, Munich, Germany). Norepinephrine was administered if the mean arterial pressure (MAP) was < 65 mmHg despite preload optimization. Dobutamine was administered if the cardiac index measured by transpulmonary thermodilution was < 2.0 L/min/m^2^ despite sufficient cardiac pre- and afterload.

### Study protocol

Patients were passively ventilated in a horizontal position with 0° body inclination throughout the study period with an Engström Carescape R860 ventilator in a volume-controlled ventilation mode using a tidal volume (*V*_T_) of 6 mL/kg of predicted body weight and a respiratory rate (RR) and fraction of inspired oxygen (FiO_2_) according to recent guidelines [19]. *V*_T_ and RR were modified only if Δ*P*_RS_ was greater than 14 cmH_2_O and pH_a_ was < 7.25.

**Fig. 1:**
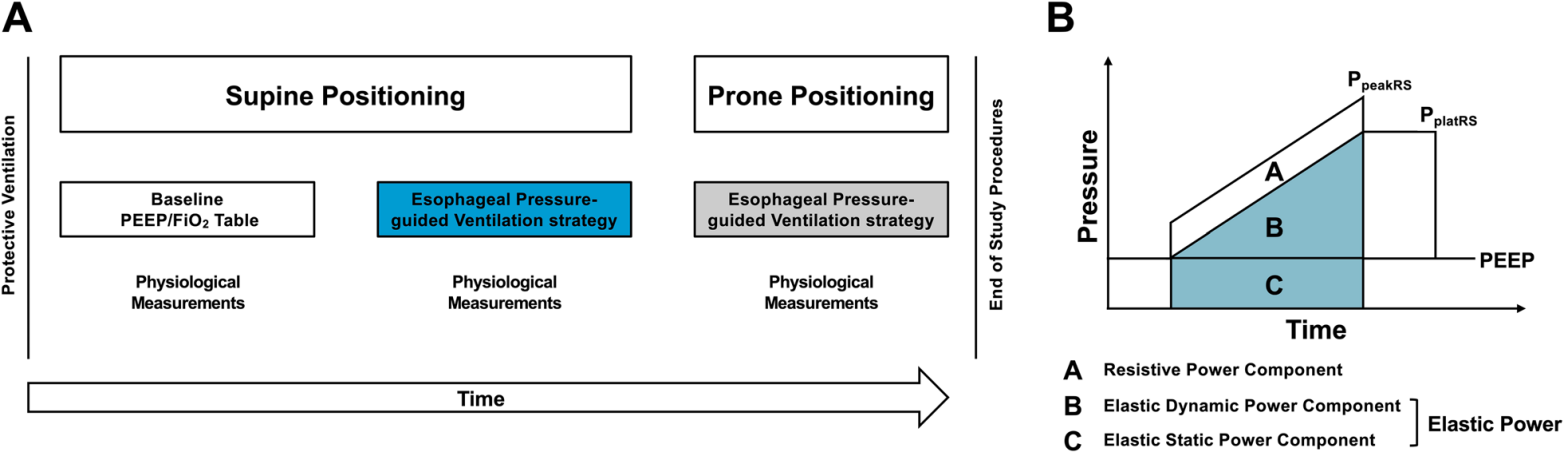
**A** Schematic workflow of the study. **B** Mechanical power components during volume-controlled ventilation with constant inspiratory flow. Area A (white) describes the resistive power component. Areas B and C describe the elastic dynamic and elastic static power components, respectively; together they are the total elastic power (teal). *P*_peakRS_, airway peak pressure; *P*_platRS_, airway plateau pressure

The study protocol included three steps (Fig. 1A): (1) baseline measurement in SP using a ventilation strategy with PEEP based on the lower PEEP/FiO_2_ table (baseline) [19, 20]; (2) P_eso_-guided ventilation strategy with PEEP targeting an end-expiratory *P*_TP_ of 0 to 2 cmH_2_O in SP [5]; (3) *P*_eso_-guided ventilation strategy with PEEP targeting an end-expiratory *P*_TP_ of 0 to 2 cmH_2_O in PP. To standardize lung volume history and allow for comparisons between ventilation strategies and positioning, a dynamic recruitment maneuver was performed before each ventilation strategy, as detailed in Additional file 1: Fig. S1. Therefore, a recruitment maneuver was performed before baseline ventilation with PEEP based on the lower PEEP/FiO_2_ table, esophageal pressure-guided PEEP in SP, and esophageal pressure-guided PEEP in PP [21].

In SP, patients were ventilated in a volume-controlled mode after the recruitment maneuver starting with a PEEP of 25 cmH_2_O. PEEP was decreased stepwise by 2 cmH_2_O every 2 min, and end-expiratory *P*_eso_ was measured during a 2-s expiratory hold. The lowest PEEP to achieve an end-expiratory *P*_TP_ of 0 to 2 cmH_2_O was used for the *P*_eso_-guided ventilation strategy (Additional file 1: Fig. S1). After completing the measurements for the *P*_eso_-guided ventilation strategy in SP, patients were placed in PP with unchanged ventilator settings. A recruitment maneuver was then performed, and PEEP was titrated to end-expiratory *P*_eso_ targeting an end-expiratory *P*_TP_ of 0 to 2 cmH_2_O. Physiologic measurements were obtained after a 30-min equilibration period with each ventilation strategy.

### Measurements

Expiratory and inspiratory airway pressures and *P*_eso_ were measured during a 2-s inspiratory and 2-s expiratory hold at zero flow to compute end-inspiratory and end-expiratory *P*_TP_ (airway pressure − *P*_eso_), respectively. Δ*P*_RS_ was calculated as airway plateau pressure (*P*_platRS_) − PEEP, and transpulmonary driving pressure (Δ*P*_TP_) as end-inspiratory *P*_TP_ − end-expiratory *P*_TP_. Static respiratory system and lung elastance were calculated as Δ*P*_RS_/*V*_T_ and Δ*P*_TP_/*V*_T_, respectively. Chest wall elastance was calculated as end-inspiratory − end-expiratory *P*_eso_/*V*_T_. The elastance ratio of the lung to the respiratory system (*E*_L_/*E*_RS_) was used to calculate lung MP components.

Lung MP was calculated as

$$0.098 \times V_{T} \times RR \times ({\text{airway}}\;{\text{peak}}\;{\text{pressure}}-\Delta P_{RS} /2) \times (E_{L} /E_{RS} )$$

Lung total elastic power was calculated as [22, 23]

$$0.098 \times V_{T} \times RR \times [(P_{{{\text{platRS}}}} + {\text{ PEEP}})/2] \times (E_{L} /E_{RS} )$$

The elastic static (related to PEEP) and dynamic (related to Δ*P*_RS_) components of lung elastic power (Fig. 1B) were calculated as

$$0.098 \times V_{{\text{T}}} \times {\text{RR}} \times {\text{ PEEP}} \times (E_{{\text{L}}} /E_{{{\text{RS}}}} )$$

and

$$0.098 \times V_{{\text{T}}} \times {\text{ RR}} \times \Delta P_{{{\text{RS}}}} /2 \times (E_{{\text{L}}} /E_{{{\text{RS}}}} )$$,

respectively [23]. Resistive power (related to the resistance of the airway) [23] was calculated as

$$0.098 \times V_{{\text{T}}} \times {\text{ RR}} \times ({\text{airway}}\;{\text{peak}}\;{\text{pressure}} -P_{{{\text{platRS}}}} )$$

Lung total elastic power and elastic static and dynamic power components were normalized to EELV to describe the energy transfer relative to the aerated lung volume (Additional file 1) [10, 11]. Lung stress was calculated using the elastance-derived method as *P*_platRS_ × (*E*_L_/*E*_RS_) [18]. EELV was measured with a modified nitrogen wash-out (20% FiO_2_ increase)/wash-in (20% FiO_2_ decrease) technique [24, 25]. At the end of the equilibration period, the alveolar dead space fraction was calculated, and arterial blood gas was analyzed. Hemodynamic parameters were obtained using transpulmonary thermodilution, and oxygen delivery was calculated. Vascular pressure transducers were zeroed to ambient pressure after changes in positioning and aligned with the phlebostatic axis corresponding to the right atrium and aortic root. For this purpose, pressure transducers were placed in the midaxillary line of the 4th intercostal space in both SP and PP to obtain comparable measurements of hemodynamic variables.

### Statistical analysis

The effect of PP on total elastic power transmitted to the lung has not been studied in patients with ARDS. Based on the results of the previous study by our group [8], an estimated sample size of 55 patients was required to measure a change in lung total elastic power (primary endpoint of the study) between SP and PP with a power of 0.8 and a significance level of 0.05. The effect size was calculated based on the change in lung total elastic power when using a *P*_eso_-guided ventilation strategy during SP and PP. Fifty-five patients underwent the current study protocol, which was part of a multipurpose study. Of these, forty patients had also been enrolled in a prior study [8], but with a different research question and study protocol. The normality of the data was tested by the Shapiro–Wilk test and subsequently analyzed using the Wilcoxon matched-pairs test for non-normally distributed data. The results are expressed as medians (interquartile range) with a level of significance set at *P* < 0.05. Statistical analyses were performed using GraphPad Prism 8 (GraphPad Software, San Diego, CA).

## Results

Fifty-five patients with moderate to severe ARDS (PaO_2_/FiO_2_ ≤ 150 mmHg) were included in the analysis. Demographic and clinical characteristics of the patients are shown in Table 1. During the study period, 48 patients required only norepinephrine, whereas one patient also required dobutamine.

**Table 1:** Demographics and clinical characteristics

Parameters	*N* = 55
Age (years)	64 (52–77)
Male sex, n (%)	37 (67)
Body mass index (kg/m^2^)	29 (26–32)
Cause of ARDS, *n* (%)	
Bacterial pneumonia	16 (29)
Viral pneumonia	28 (51)
Non-pulmonary sepsis	11 (20)
Mechanical ventilation before study (days)	4 (3–6)
SOFA	10 (9–12)
SAPS II	70 (63–78)
Length of ICU stay (days)	17 (11–29)
ICU mortality, n (%)	20 (36)

Data are shown as medians (interquartile range) or numbers (%)

*ARDS* acute respiratory distress syndrome, *SOFA* sequential organ failure assessment score, *SAPS II* simplified acute physiology score, *ICU* intensive care unit

### Effects of prone positioning

PP resulted in lower PEEP and *P*_platRS_ compared with SP, but end-inspiratory and end-expiratory *P*_TP_ as well as EELV did not differ by positioning (Table 2).

**Table 2:** Effects of supine and prone positioning on respiratory parameters

Parameters	Baseline	Supine	Prone	*P* values		
	PEEP/FiO_2_ table	*P*_eso_-guided	*P*_eso_-guided	Supine versus Baseline	Prone versus Baseline	**Prone versus** **Supine**
Tidal volume (mL/kg PBW)	6.1 (6.0 to 6.2)	6.1 (6.0 to 6.2)	6.1 (6.0 to 6.2)	> 0.999	> 0.999	> 0.999
Respiratory rate (breaths/min)	23 (21 to 24)	23 (21 to 24)	23 (21 to 24)	> 0.999	> 0.999	> 0.999
PEEP (cmH_2_O)	8 (8 to 10)	15 (12 to 18)	10 (7 to 15)	< 0.001	0.003	< 0.001
Airway peak pressure (cmH_2_O)	25 (19 to 28)	30 (25 to 35)	26 (21 to 32)	< 0.001	0.001	< 0.001
Airway plateau pressure (cmH_2_O)	18 (16 to 22)	25 (20 to 27)	20 (16 to 26)	< 0.001	0.004	< 0.001
Driving pressure (cmH_2_O)	9 (8 to 11)	9 (7 to 11)	9 (8 to 11)	0.187	0.835	0.076
End-expiratory *P*_eso_ (cmH_2_O)	12 (9 to 14)	14 (9 to 16)	10 (6 to 12)	< 0.001	< 0.001	< 0.001
End-inspiratory *P*_eso_ (cmH_2_O)	15 (12 to 17)	17 (12 to 21)	14 (10 to 19)	< 0.001	0.048	< 0.001
End-expiratory *P*_TP_ (cmH_2_O)	− 2 (− 5 to 0)	1 (0 to 2)	1 (0 to 2)	< 0.001	< 0.001	0.650
End-inspiratory *P*_TP_ (cmH_2_O)	3 (1 to 7)	8 (4 to 9)	7 (5 to 9)	< 0.001	< 0.001	0.495
Driving *P*_TP_ (cmH_2_O)	6 (4 to 8)	6 (4 to 7)	5 (3 to 7)	0.029	< 0.001	0.117
Respiratory system elastance (cmH_2_O/L)	20.0 (16.8 to 29.5)	18.9 (16.1 to 27.3)	22.2 (17.8 to 24.9)	0.049	0.448	0.124
Lung elastance (cmH_2_O/L)	14.5 (10.7 to 18.8)	13.3 (8.7 to 17.0)	12.6 (8.1 to 18.5)	0.040	0.002	0.122
Chest wall elastance (cmH_2_O/L)	6.9 (4.9 to 11.2)	7.3 (4.9 to 10.5)	10.8 (5.2 to 13.5)	0.909	< 0.001	< 0.001
*E*_L_/*E*_RS_	0.67 (0.56 to 0.77)	0.67 (0.50 to 0.78)	0.61 (0.38 to 0.73)	0.189	< 0.001	< 0.001
EELV (L)	1.5 (1.2 to 2.0)	2.1 (1.6 to 2.6)	2.1 (1.6 to 2.7)	< 0.001	< 0.001	0.843
Lung stress (cmH_2_O)	12.3 (9.1 to 15.0)	15.2 (10.7 to 19.8)	11.0 (7.4 to 14.1)	< 0.001	0.074	< 0.001
Resistive power (J/min)	4.4 (3.3 to 6.8)	4.7 (3.5 to 6.4)	4.7 (3.6 to 7.7)	0.892	0.045	0.060
Lung MP (J/min)	11.6 (8.0 to 15.5)	13.9 (9.1 to 19.9)	9.9 (6.5 to 15.7)	< 0.001	0.070	< 0.001
Lung MP normalized to EELV (J/min/L)	6.8 (5.1 to 10.0)	6.8 (4.2 to 9.7)	4.6 (2.9 to 7.0)	0.262	< 0.001	< 0.001

Data are shown as medians (interquartile range). A ventilation strategy with PEEP based on the PEEP/FiO_2_ table was used as the baseline. For the comparison between supine and prone positioning, a ventilation strategy with esophageal pressure-guided PEEP was used. Pairwise comparisons were made using the Wilcoxon matched-pairs test. PBW, predicted body weight; PEEP, positive end-expiratory pressure; FiO_2_, fraction of inspired oxygen; *P*_eso_, esophageal pressure; *P*_TP_, transpulmonary pressure; *E*_L_/*E*_RS_, elastance ratio of the lung to the respiratory system; EELV, end-expiratory lung volume; MP, mechanical power

**Fig. 2:**
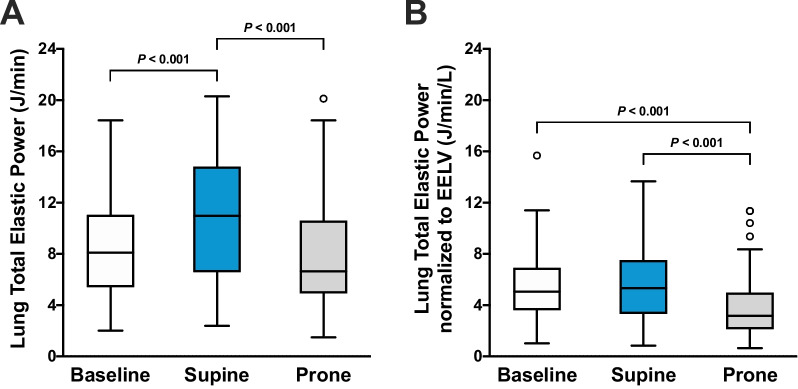
Effects of supine and prone positioning on **A** lung total elastic power and **B** lung total elastic power normalized to end-expiratory lung volume (EELV). A ventilation strategy with positive end-expiratory pressure (PEEP) based on the PEEP/FiO_2_ table during supine positioning was used as the baseline. A ventilation strategy with esophageal pressure-guided PEEP was used for the comparison between supine and prone positioning. Boxplots show the interquartile range and median with whiskers according to Tukey's method. Outliers are shown as circles. Brackets denote statistically significant differences between positioning and ventilation strategies; *P* values are shown above the brackets

Lung total elastic power was lower during PP compared with SP (6.7 [4.9–10.6] versus 11.0 [6.6–14.8] J/min; *P* < 0.001) (Fig. 2A). PP also resulted in lower lung total elastic power normalized to EELV compared with SP (3.2 [2.1–5.0] versus 5.3 [3.3–7.5] J/min/L; *P* < 0.001) (Fig. 2B).

**Fig. 3:**
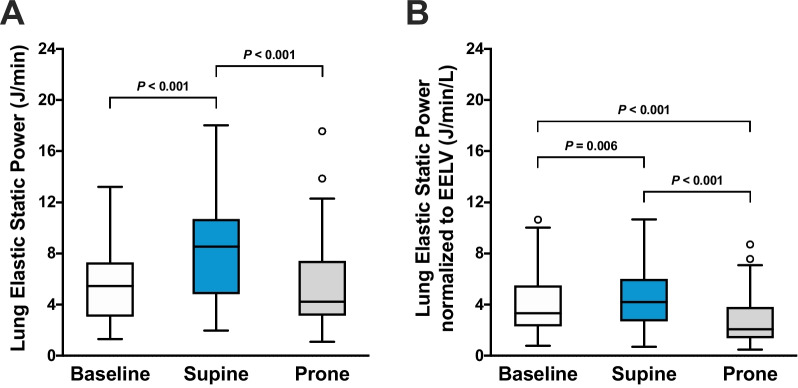
Effects of supine and prone positioning on **A** lung elastic static power and **B** lung elastic static power normalized to end-expiratory lung volume (EELV). A ventilation strategy with positive end-expiratory pressure (PEEP) based on the PEEP/FiO_2_ table during supine positioning was used as the baseline. A ventilation strategy with esophageal pressure-guided PEEP was used for the comparison between supine and prone positioning. Boxplots show the interquartile range and median with whiskers according to Tukey's method. Outliers are shown as circles. Brackets denote statistically significant differences between positioning and ventilation strategies; *P* values are shown above the brackets

**Fig. 4:**
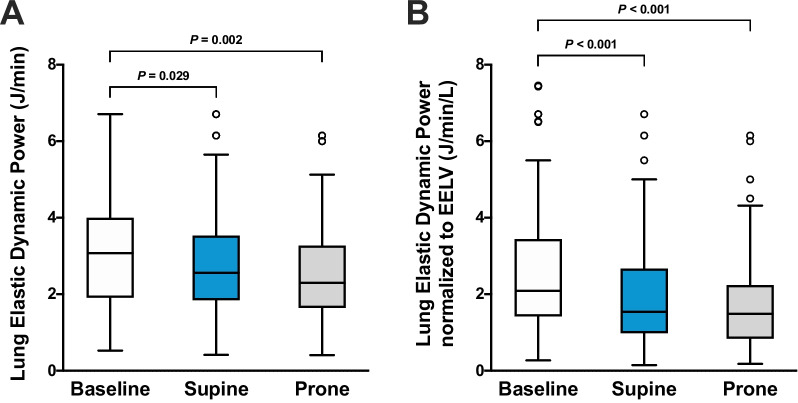
Effects of supine and prone positioning on **A** lung elastic dynamic power and **B** lung elastic dynamic power normalized to end-expiratory lung volume (EELV). A ventilation strategy with positive end-expiratory pressure (PEEP) based on the PEEP/FiO_2_ table during supine positioning was used as the baseline. A ventilation strategy with esophageal pressure-guided PEEP was used for the comparison between supine and prone positioning. Boxplots show the interquartile range and median with whiskers according to Tukey's method. Outliers are shown as circles. Brackets denote statistically significant differences between positioning and ventilation strategies; *P* values are shown above the brackets

PP reduced lung elastic static power compared with SP (4.2 [3.2–7.4] versus 8.5 [4.8–10.7] J/min; *P* < 0.001) (Fig. 3A) and lung elastic static power normalized to EELV (2.1 [1.4–3.8] versus 4.2 [2.7–6.0] J/min/L; *P* < 0.001) (Fig. 3B). PP did not affect lung elastic dynamic power non-normalized and normalized to EELV (Fig. 4A, B). Compared with SP, PP reduced *E*_L_/*E*_RS_ and lung stress and increased chest wall elastance when using *P*_eso_-guided ventilation (Table 2).

PaO_2_/FiO_2_ increased and the shunt fraction and the alveolar dead space fraction decreased during PP compared with SP with *P*_eso_-guided ventilation (Table 3). PP resulted in higher MAP and cardiac output and increased oxygen delivery compared with SP. Further details regarding the effect of PP on respiratory parameters, gas exchange, and hemodynamic parameters are shown in Tables 2, 3, and Additional file 1: Tables S2 and S3.

**Table 3:** Effects of supine and prone positioning on gas exchange and hemodynamic parameters

Parameters	Baseline	Supine	Prone	*P* values		
	PEEP/FiO_2_ table	*P*_eso_-guided	*P*_eso_-guided	Supine versus Baseline	Prone versus Baseline	Prone versus Supine
PaO_2_/FiO_2_ (mmHg)	124 (110 to 154)	176 (144 to 237)	222 (177 to 299)	< 0.001	< 0.001	< 0.001
Shunt fraction (%)	47 (36 to 53)	33 (24 to 40)	28 (21 to 39)	< 0.001	< 0.001	0.009
PaCO_2_ (mmHg)	55 (48 to 62)	56 (51 to 62)	57 (50 to 62)	0.528	0.592	0.955
pHa	7.3 (7.3 to 7.4)	7.3 (7.3 to 7.4)	7.3 (7.3 to 7.4)	0.709	0.715	0.335
Alveolar dead space fraction	0.28 (0.22 to 0.37)	0.29 (0.22 to 0.37)	0.26 (0.20 to 0.34)	0.989	0.003	0.001
Heart rate (bpm)	90 (78 to 106)	91 (76 to 105)	92 (79 to 106)	0.291	0.393	0.448
Mean arterial pressure (mmHg)	81 (76 to 86)	79 (71 to 87)	87 (77 to 95)	0.024	0.003	< 0.001
Central venous pressure (mmHg)	14 (11 to 17)	17 (12 to 19)	17 (12 to 22)	< 0.001	0.001	0.207
Norepinephrine (μg/kg/min)	0.09 (0.03 to 0.26)	0.09 (0.04 to 0.27)	0.07 (0.03 to 0.29)	0.417	0.196	0.133
Cardiac output (L/min)	7.1 (5.6 to 8.3)	6.1 (5.1 to 7.8)	7.0 (5.6 to 8.3)	< 0.001	0.809	< 0.001
Oxygen delivery (mL/min)	878 (687 to 1078)	809 (685 to 1047)	915 (720 to 1206)	0.133	< 0.001	< 0.001

Data are shown as medians (interquartile range). A ventilation strategy with PEEP based on the PEEP/FiO_2_ table was used as the baseline. For the comparison between supine and prone positioning, a ventilation strategy with esophageal pressure-guided PEEP was used. During the study period, norepinephrine was required in 48 patients. Pairwise comparisons were made using the Wilcoxon matched-pairs test. PEEP, positive end-expiratory pressure; FiO_2_, fraction of inspired oxygen; *P*_eso_, esophageal pressure; PaO_2_/FiO_2_, arterial partial pressure of oxygen divided by fraction of inspired oxygen; PaCO_2_, arterial partial pressure of carbon dioxide; pHa, negative logarithm of the molar concentration of dissolved hydronium ions in arterial blood

### Effects of ventilation strategies during supine positioning

*P*_eso_-guided ventilation in SP resulted in higher PEEP and *P*_platRS_ compared with an oxygenation-guided ventilation strategy using the PEEP/FiO_2_ table (baseline). This increased end-inspiratory and end-expiratory *P*_TP_ as well as EELV (Table 2).

*P*_eso_-guided ventilation during SP compared with baseline increased lung total elastic power (11.0 [6.6–14.8] versus 8.1 [5.4–11.0] J/min; *P* < 0.001) (Fig. 2A), but lung total elastic power normalized to EELV did not differ according to the ventilation strategy during SP (5.3 [3.3–7.5] versus 5.1 [3.6–6.9] J/min/L; *P* = 0.538) (Fig. 2B). Lung elastic static power was higher with *P*_eso_-guided ventilation during SP compared with baseline (8.5 [4.8–10.7] versus 5.5 [3.1–7.3] J/min; *P* < 0.001) (Fig. 3A). Similarly, *P*_eso_-guided ventilation increased lung elastic static power normalized to EELV compared with baseline (4.2 [2.7–6.0] versus 3.3 [2.3–5.5] J/min/L; *P* = 0.006) (Fig. 3B). *P*_eso_-guided ventilation during SP compared with baseline decreased Δ*P*_TP_, lung elastic dynamic power (2.5 [1.8–3.5] versus 2.9 [1.9–3.7] J/min; *P* = 0.029) (Fig. 4A) and lung elastic dynamic power normalized to EELV (1.2 [0.7–1.9] versus 1.7 [1.3–2.7] J/min/L; *P* < 0.001) (Fig. 4B), but lung stress was higher (Table 2).

PaO_2_/FiO_2_ increased and the shunt fraction decreased with *P*_eso_-guided ventilation in SP compared with baseline (Table 3). *P*_eso_-guided ventilation resulted in lower MAP and cardiac output during SP compared with baseline but did not affect oxygen delivery (Table 3**)**.

## Discussion

In this prospective, physiologic study on the effect of PP combined with protective ventilation on lung total elastic power in fifty-five patients with moderate to severe ARDS, we found that: (A) PP reduced lung total elastic power and lung total elastic power normalized to EELV compared with SP; (B) lung elastic static power and lung elastic static power normalized to EELV were lower in PP compared with SP using *P*_eso_-guided ventilation because comparable *P*_TP_ and EELV were achieved at lower airway pressures; (C) PP did not reduce lung elastic dynamic power with *P*_eso_-guided ventilation compared with SP regardless of EELV normalization; (D) PP improved gas exchange and hemodynamics, thus mitigating the adverse effects of higher airway pressures associated with *P*_eso_-guided ventilation while optimizing oxygen delivery.

To the best of our knowledge, this is the first study prospectively investigating the physiologic effects of PP on lung-transmitted static and dynamic MP components (excluding MP transmission to the chest wall). Normalization of static and dynamic MP components to EELV, which surrogates energy transfer per aerated lung volume, may further enhance the relevance of the present data.

### Effects of prone positioning

Total MP, which combines static and dynamic parameters of ventilation, has been investigated to quantify the invasiveness of ventilation and may be related to the risk of VILI [26, 27]. Thus, the present study compared elastic power and its components (static and dynamic), to estimate lung stress and strain, according to different positioning and ventilation strategies [22].

However, the role of MP, its static and dynamic components, and the relevance in comparison with simpler bedside indices, e.g., 4 × Δ*P*_RS_ + RR, or any other predictor of VILI remains unclear [23, 28]. Despite the debate about the importance of each MP component, high MP per se, which combines different ventilator variables, increases the risk of VILI in patients with ARDS. Moreover, reducing only one variable may be insufficient to significantly modify MP [26]. MP, compared to single-ventilator variables or simpler indices, may thus provide a holistic picture on the invasiveness of different ventilation strategies and the effect of positioning. Normalizing MP transfer to the size of the aerated lung may be the essential step for the clinical use of MP and definition of safety thresholds [11, 27]. This has been shown to correlate power transfer with lung stress and strain [29] and further improve the prediction of mortality in patients with ARDS [30]. However, estimating energy transfer per aerated lung volume in clinical practice may be hindered by the requirement to measure EELV. Although normalization to body weight or compliance has been suggested, the optimal method remains unclear [27].

Another method to measure global lung stress is to isolate the fraction of airway pressure applied to the lung (*P*_TP_) using *P*_eso_ measurements [5]. This can provide relevant information regarding the invasiveness of ventilation in situations with altered chest wall mechanics such as PP [8, 14, 31]. In this situation, *P*_eso_-guided ventilation with PEEP titrated to maintain a positive end-expiratory *P*_TP_ may be clinically useful to balance lung recruitment and overdistension in patients with ARDS [32].

Thus, utilizing the *P*_eso_ measurement to quantify lung MP and normalizing to EELV may add important information regarding the invasiveness of ventilation in patients with ARDS managed with PP, because PP modifies chest wall elastance and increases EELV and lung homogeneity [27, 33]. We modified PEEP in PP to account for the reduced vertical pleural pressures and the accompanying regional changes in lung mechanics in each individual patient [7, 12–14, 34] and to avoid the influence of inadequate (excessive or insufficient) PEEP on total elastic power transmitted to the lung [6]. In our study, *V*_T_ and RR were comparable between ventilation strategies and positioning; thus, changes in lung total elastic power and its components were due to changes in respiratory mechanics addressed by individualized ventilation strategies.

A recent study by Morais et al. evaluated respiratory mechanics in SP and PP over a range of PEEP levels in patients with ARDS and found a variety of responses in global and regional mechanics induced by PP, suggesting the need to individualize PEEP according to the positioning [35]. On the contrary, a study by Mezidi et al. found no significant differences in PEEP titrated according to end-expiratory *P*_TP_ when patients were turned from SP to PP [36]. Of note and in contrast to our study with 0° body inclination for both SP and PP, the study compared SP with 30° to PP with 0° to 15° body inclination [36]. Body inclination has been shown to affect respiratory mechanics and EELV in mechanically ventilated patients with ARDS due to changes in chest wall elastance and *P*_TP_ [37, 38].

The effect of PEEP in patients with ARDS is critically dependent on lung recruitability [39]; however, the large vertical pleural pressure gradient present in supine patients with ARDS [17] may not allow for significant recruitment without concomitant overdistension due to differences in regional *P*_TP_ [40]. In our study, PP resulted in a significant reduction of lung total elastic power and lung total elastic power normalized to EELV compared with SP. Although the role of static and dynamic MP components in the pathogenesis of VILI is debated [23, 28], excessive MP, regardless of the constituents, causes similar lung injury [26, 27]. PP decreases pleural pressure gradients and homogenizes ventilation [7], reducing the risk of VILI [4, 41] by limiting regional lung strain due to overdistension and tidal recruitment [3].

In moderate to severe ARDS patients with recruitable lung parenchyma, PP increases end-expiratory *P*_TP_ and EELV [8, 39]. As demonstrated in the present physiologic study, PP may therefore be a part of a lung-protective ventilation strategy aimed at reducing lung total elastic power transmission per aerated lung volume. This may reduce damaging ventilation above the proposed parenchymal stress threshold by decreasing lung strain while increasing the size of the aerated lung [10, 42]**.** Our results expand the mechanistic understanding of the effects of PP to improve lung protection by reducing energy transfer per aerated lung volume, which has been discussed as a major factor for improved survival in the PROSEVA trial [2–4].

On the other hand, in our study, PP did not reduce lung elastic dynamic power non-normalized and normalized to EELV with *P*_eso_-guided ventilation. The dynamic component of lung MP is exponentially affected by *V*_T_ [9] and may lead to increased inspiratory lung strain, as indicated by the resulting Δ*P*_TP_ [43]. High MP due to excessive *V*_T_ causing damaging lung stress and strain is clinically indicated by altered respiratory mechanics with sharply increased *P*_platRS_ and Δ*P*_TP_ when EELV is kept constant [26]. Although the decrease in Δ*P*_TP_ and lung elastance with comparable EELV during PP compared to SP was non-significant, this trend may signify the opening of new lung units and/or improved mechanical properties of previously ventilated lung units [44]. Consequently, *V*_T_ is evenly distributed between dependent and non-dependent lung regions during PP and overdistension is limited [7], as reflected by reduced end-inspiratory lung stress and total elastic power normalized to EELV. Prolonged periods of PP may further reduce total elastic power transfer per aerated lung volume, as EELV has been shown to increase over time in PP [36].

The key message of our study for clinical practice is that PP allows for a reduction in lung elastic power transmission per aerated lung volume because comparable *P*_TP_ and EELV can be achieved at lower airway pressures. This may have important implications for the ventilator management during PP.

### Effects of ventilation strategies during supine positioning

In our study, compared with baseline, *P*_eso_-guided ventilation in SP resulted in higher PEEP, *P*_platRS_, and EELV, thereby improving PaO_2_/FiO_2_ due to a reduced pulmonary shunt. However, this ventilation strategy increased lung total elastic power and lung stress. This is consistent with the results of the EPVent-2 trial, where *P*_eso_-guided ventilation resulted in PEEP levels similar to ours and did not reduce Δ*P*_TP_ compared with a ventilation strategy using the higher PEEP/FiO_2_ table [45]. Although there was no significant difference in lung total elastic power normalized to EELV when using *P*_eso_-guided ventilation in SP compared with baseline, higher PEEP and *P*_platRS_ may have resulted in overdistension of non-dependent lung regions, despite improving dependent lung aeration by maintaining positive end-expiratory *P*_TP_ and increasing EELV [18]. Our study demonstrates that PP compared to SP can offset the need for higher airway pressures to maintain positive end-expiratory *P*_TP_ and reduce pulmonary shunt. This results in a reduction in total elastic and elastic static power transmission per aerated lung volume compared to SP.

Theoretically, the overall effect of a reduction in end-expiratory *P*_TP_ on lung MP is less pronounced than the effect of changes in *V*_T_, *Δ*P_RS_, and inspiratory airflow [9]. This suggests that reducing elastic static power may have a minor impact on lung protection; however, a U-shaped relationship between end-expiratory *P*_TP_ and the risk of VILI has been discussed, with both insufficient and excessive end-expiratory *P*_TP_ causing VILI due to atelectrauma and overdistension, respectively [22, 26, 32, 46]. As shown experimentally, the application of inadequate lung elastic static power can impair lung structural architecture and elastance, and increase extravascular lung water and inflammation [26, 46]. Furthermore, the combination of elastic static and dynamic power, but not elastic static or dynamic power alone, correlated with alveolar collapse and regional overdistension as hallmarks of VILI [22]. This highlights the importance of not reaching a critical lung stress and strain threshold [10].

In SP, *P*_eso_-guided ventilation with higher PEEP and *P*_platRS_ resulted in a reduction of cardiac output in comparison with baseline. The results of our study are consistent with the findings in an animal model, where higher elastic static power caused severe hemodynamic impairment [26], which could be translated to the clinical setting. Adverse hemodynamic effects of mechanical ventilation are common in patients with ARDS [47], but may be limited by PP [48]. PP with *P*_eso_-guided ventilation restored cardiac output and increased MAP and oxygen delivery in comparison with SP. Possible mechanisms for this effect of PP in our study may include an increased gradient for venous return by increasing intra-abdominal pressure and reduced lung overdistension with decreased pulmonary vascular resistance and right ventricular afterload, thereby improving right ventricular function [47–49].

### Clinical Implications

PP improves survival in patients with moderate to severe ARDS, possibly by improving lung protection and reducing VILI [2–4]. This might be due to a reduction in the pleural pressure gradient with changes in *P*_TP_ and EELV that affect the transmission of static and dynamic MP components. The short-term physiologic data from our study suggest that PP may allow for a reduction in lung elastic power without a loss of EELV, thereby minimizing energy transfer per aerated lung volume and improving gas exchange, hemodynamics, and oxygen delivery in patients with moderate to severe ARDS. Furthermore, quantifying lung MP and normalizing it to EELV may be an essential step to describe the energy transfer relative to the aerated lung volume at the bedside to better monitor mechanically ventilated patients with ARDS.

### Limitations

Our study has several limitations. We studied the short-term physiologic effects of PP using *P*_eso_-guided ventilation with similar *per protocol* *V*_T_ and RR; thus, we cannot exclude that the effects of PP on lung MP components differ according to ventilation strategy. In line with the mechanistic understanding of VILI, maintaining a positive end-expiratory *P*_TP_ has been associated with lower mortality in a post hoc re-analysis of the EPVent-2 trial [5]; however, the optimal ventilation strategy during PP is unclear.

Individual lung recruitability was not assessed before the study, and the effect of PEEP on lung total elastic power, lung elastic static, and dynamic power components, as well as respiratory and hemodynamic parameters, may depend on recruitability [50]. During protective ventilation, a U-shaped relationship between PEEP and VILI has been suggested by experimental studies [22, 26, 46], but the best method to individualize PEEP is unknown [19].

Our study focused on lung total elastic power including its elastic static and dynamic components to approximate lung stress and strain [22]. We excluded the resistive component of MP [9] because the biological impact of this component in comparison with the elastic power components is unclear [10, 11, 51]. Additionally, estimating energy transfer per aerated lung volume by normalizing MP in clinical practice may be hindered by the requirement to measure EELV, and the optimal method to normalize MP is unclear [27].

Another limitation is the lack of an imaging technology, e.g., electrical impedance tomography, to quantify regional lung aeration, as the physiologic effects of PP may be heterogeneous [35]. Although lung total elastic power, power components normalized to EELV, and lung stress were minimized, we cannot formally exclude regional hyperinflation in PP.

## Conclusions

In patients with moderate to severe ARDS, compared with SP, PP reduced lung total elastic and elastic static power, minimizing total elastic and elastic static power transmission per aerated lung volume, because comparable values for *P*_TP_ and EELV were achieved at lower airway pressures. This resulted in improved gas exchange, hemodynamics, and oxygen delivery. Due to the changes in chest wall mechanics and lung aeration during pronation, quantifying lung MP and normalizing it to EELV may be an essential step to describe the energy transfer relative to the aerated lung volume and further understand the lung-protective effect of PP.

### Supplementary Information

**Additional file 1**. Study details and equations for physiologic variables.

## Data Availability

The datasets analyzed during this study are available from the corresponding author on reasonable request.

## Abbreviations

Δ*P*_RS_: Respiratory system driving pressure
Δ*P*_TP_: Transpulmonary driving pressure
ARDS: Acute respiratory distress syndrome
EELV: End-expiratory lung volume
*E*_L_/*E*_RS_: Elastance ratio of the lung to the respiratory system
FiO_2_: Fraction of inspired oxygen
ICU: Intensive care unit
MAP: Mean arterial pressure
MP: Mechanical power
PaO_2_/FiO_2_: Ratio of arterial partial pressure of oxygen to the fraction of inspired oxygen
PEEP: Positive end-expiratory pressure
*P*_eso_: Esophageal pressure
pHa: Negative logarithm of the molar concentration of dissolved hydronium ions in arterial blood
*P*_peakR__S_: Airway peak pressure
*P*_platRS_: Airway plateau pressure
PP: Prone positioning
*P*_TP_: Transpulmonary pressure
RR: Respiratory rate
SP: Supine positioning
SAPS II: Simplified Acute Physiology Score
SOFA: Sequential Organ Failure Assessment
VILI: Ventilator-induced lung injury
*V*_T_: Tidal volume
