# Supplementary material for: Effects of prone positioning on lung mechanical power components in patients with acute respiratory distress syndrome: a physiologic study
Source: Crit Care. 2024 Mar 15;28:82. doi: 10.1186/s13054-024-04867-6 (PMC10941550; doi:10.1186/s13054-024-04867-6)
Supplement: Supplementary file 1 — Additional file 1. Study details and equations for physiologic variables. [file 13054_2024_4867_MOESM1_ESM.docx]

Effects of prone positioning on lung mechanical power components in patients with acute respiratory distress syndrome: a physiologic study

**Table S1** Lower PEEP/FiO_2_ table according to the ARDS Network [1]: allowable combinations of PEEP and FiO_2_

| FiO_2_ (%) | 30 | 40 | 40 | 50 | 50 | 60 | 70 | 70 | 70 | 80 | 90 | 90 | 90 | 100 |
| --- | --- | --- | --- | --- | --- | --- | --- | --- | --- | --- | --- | --- | --- | --- |
| PEEP (cmH_2_O) | 5 | 5 | 8 | 8 | 10 | 10 | 10 | 12 | 14 | 14 | 14 | 16 | 18 | 18–24 |

PEEP and FiO_2_ were titrated to achieve an arterial oxygen saturation of 88%–92%. Adapted from [1].

FiO_2_, fraction of inspired oxygen; PEEP, positive end-expiratory pressure.


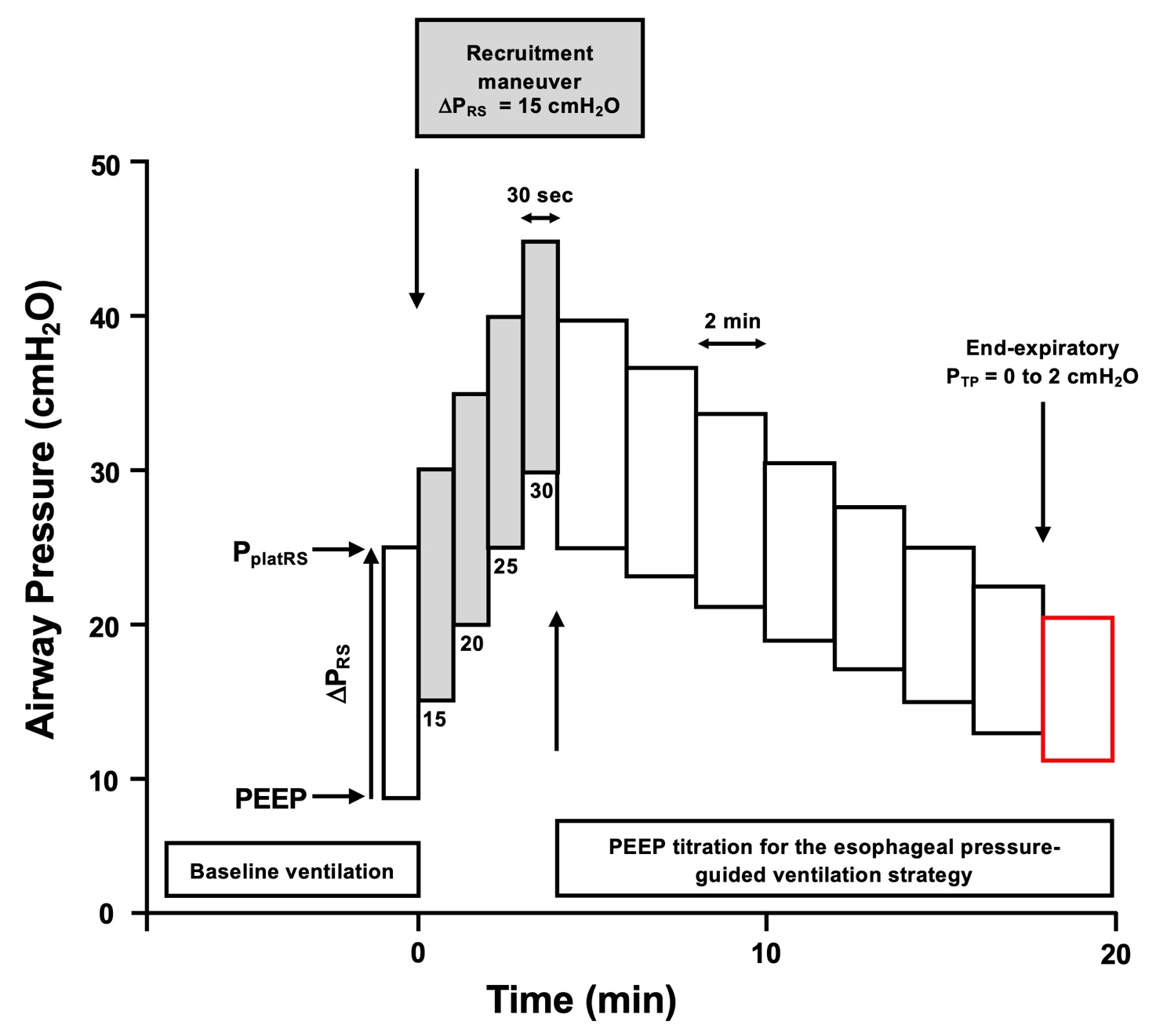


**Fig. S1** Schematic representation of the dynamic recruitment maneuver. ΔP_RS_, respiratory system driving pressure; PEEP, positive end-expiratory pressure; P_platRS_, respiratory system airway plateau pressure; P_TP_, transpulmonary pressure.

To standardize lung volume history and allow for comparisons between ventilation strategies and positioning, a dynamic recruitment maneuver was performed before each of the three experimental steps [2]. For this purpose, PEEP was increased gradually to 30 cmH_2_O in a pressure-controlled mode with a driving pressure (ΔP_RS_) of 15 cmH_2_O. After 30 s, the ventilator was switched back to a volume-controlled mode starting with a PEEP of 25 cmH_2_O. PEEP was decreased stepwise by 2 cmH_2_O every 2 min and end-expiratory esophageal pressure was measured during a 2-s expiratory hold. The lowest PEEP to achieve an end-expiratory transpulmonary pressure of 0 to 2 cmH_2_O was used for the esophageal pressure-guided ventilation strategy. Following prone positioning, the procedure was repeated, and PEEP was titrated to end-expiratory esophageal pressure targeting an end-expiratory transpulmonary pressure of 0 to 2 cmH_2_O.

Before performing the recruitment maneuver, vasopressor infusion was increased to achieve a mean arterial pressure ≥75 mmHg and the recruitment maneuver was terminated in the case of hemodynamic instability (defined as mean arterial pressure <65 mmHg or cardiac output <2.0 L/min/m^2^ despite vasopressor treatment).

# Equations for physiologic variables

Alveolar dead space fraction = (arterial partial pressure of carbon dioxide (PaCO_2_) − mean partial pressure of exhaled CO_2_)/PaCO_2_

Elastance ratio of the lung to the respiratory system = E_L_/E_RS_

End-expiratory P_TP_ = PEEP − end-expiratory P_eso_

End-inspiratory transpulmonary pressure (P_TP_) = end-inspiratory airway plateau pressure − end-inspiratory esophageal pressure (P_eso_)

Lung elastic dynamic power = 0.098 × V_T_ × RR × ΔP_RS_/2 × (E_L_/E_RS_)

Lung elastic dynamic power normalized to EELV = lung elastic dynamic power/EELV

Lung elastic static power = 0.098 × V_T_ × RR × PEEP × (E_L_/E_RS_)

Lung elastic static power normalized to EELV = lung elastic static power/EELV

Lung mechanical power = 0.098 × V_T_ × RR × (P_peakRS_ − ΔP_RS_/2) × (E_L_/E_RS_)

Lung mechanical power normalized to EELV = lung mechanical power/EELV

Lung stress = P_platRS_ × (E_L_/E_RS_)

Lung total elastic power = 0.098 × V_T_ × RR × ([P_platRS_ + PEEP]/2) × (E_L_/E_RS_)

Lung total elastic power normalized to EELV = lung total elastic power/EELV

Oxygen delivery = cardiac output × 1.34 × hemoglobin × arterial oxygen saturation + (0.003 × arterial partial pressure of oxygen)

Resistive power = 0.098 × V_T_ × RR × (P_peakRS_ − P_platRS_)

Respiratory system driving pressure (ΔP_RS_) = airway plateau pressure (P_platRS_) − PEEP

Shunt fraction = (alveolar oxygen content − arterial oxygen content)/(alveolar oxygen content − venous oxygen content)

Static chest wall elastance = (end-inspiratory P_eso_ − end-expiratory P_eso_)/V_T_

Static lung elastance (E_L_) = ΔP_TP_/V_T_

Static respiratory system elastance (E_RS_) = ΔP_RS_/V_T_

Transpulmonary driving pressure (ΔP_TP_) = end-inspiratory P_TP_ − end-expiratory P_TP_

**Table S2** Effects of supine and prone positioning on respiratory parameters

|  | **Difference (95% CI)** | | | ***P* values** | | |
| --- | --- | --- | --- | --- | --- | --- |
| **Parameters** | **Supine versus Baseline** | **Prone versus**  **Baseline** | **Prone versus**  **Supine** | **Supine versus Baseline** | **Prone versus**  **Baseline** | **Prone versus**  **Supine** |
| Tidal volume (mL/kg PBW) | 0.0 (0.0 to 0.0) | 0.0 (0.0 to 0.0) | 0.0 (0.0 to 0.0) | >0.999 | >0.999 | >0.999 |
| Respiratory rate (breaths/min) | 0 (0 to 0) | 0 (0 to 0) | 0 (0 to 0) | >0.999 | >0.999 | >0.999 |
| PEEP (cmH_2_O) | 6 (5 to 8) | 2 (0 to 3) | −4 (−5 to −3) | <0.001 | 0.003 | <0.001 |
| Airway peak pressure (cmH_2_O) | 6 (4 to 7) | 3 (1 to 4) | −3 (−4 to −2) | <0.001 | 0.001 | <0.001 |
| Airway plateau pressure (cmH_2_O) | 6 (4 to 7) | 2 (1 to 3) | −3 (−5 to −2) | <0.001 | 0.004 | <0.001 |
| Driving pressure (cmH_2_O) | 0 (−1 to 0) | 0 (0 to 1) | 1 (0 to 1) | 0.187 | 0.835 | 0.076 |
| End-expiratory P_eso_ (cmH_2_O) | 2 (1 to 3) | −2 (−3 to −1) | −4 (−5 to −3) | <0.001 | <0.001 | <0.001 |
| End-inspiratory P_eso_ (cmH_2_O) | 2 (1 to 3) | −0.5 (−3 to 0) | −3 (−5 to −2) | <0.001 | 0.048 | <0.001 |
| End-expiratory P_TP_ (cmH_2_O) | 4 (3 to 5) | 4 (3 to 6) | 0 (0 to 0) | <0.001 | <0.001 | 0.650 |
| End-inspiratory P_TP_ (cmH_2_O) | 4 (2 to 5) | 4 (3 to 5) | 0 (−1 to 0) | <0.001 | <0.001 | 0.495 |
| Driving P_TP_ (cmH_2_O) | 0 (−1 to 0) | −1 (−2 to 0) | 0 (−1 to 0) | 0.029 | <0.001 | 0.117 |
| Respiratory system elastance (cmH_2_O/L) | 0.0 (−2.3 to 0.0) | 0.0 (0.0 to 0.0) | 2.1 (0.0 to 2.6) | 0.049 | 0.448 | 0.124 |
| Lung elastance (cmH_2_O/L) | 0.0 (−2.2 to 0.0) | −2.3 (−4.2 to 0.0) | 0.0 (−2.2 to 0.0) | 0.040 | 0.002 | 0.122 |
| Chest wall elastance (cmH_2_O/L) | 0.0 (0.0 to 0.0) | 2.2 (0.0 to 2.8) | 2.3 (2.0 to 3.0) | 0.909 | <0.001 | <0.001 |
| E_L_/E_RS_ | −0.01 (−0.06 to 0.0) | −0.09 (−0.14 to −0.04) | −0.07 (−0.10 to −0.02) | 0.189 | <0.001 | <0.001 |
| EELV (L) | 0.4 (0.3 to 0.5) | 0.4 (0.2 to 0.7) | 0.0 (−0.1 to 0.1) | <0.001 | <0.001 | 0.843 |
| Lung stress (cmH_2_O) | 2.5 (1.3 to 3.6) | −1.0 (−2.3 to 0.0) | −2.8 (−4.0 to −2.1) | <0.001 | 0.074 | <0.001 |
| Resistive power (J/min) | 0.0 (−0.6 to 0.0) | 0.0 (0.0 to 0.9) | 0.0 (0.0 to 0.6) | 0.892 | 0.045 | 0.060 |
| Lung MP (J/min) | 2.1 (1.2 to 3.2) | −0.7 (−1.6 to 0.0) | −2.4 (−4.0 to −1.6) | <0.001 | 0.070 | <0.001 |
| Lung MP normalized to EELV (J/min/L) | −0.1 (−0.8 to 0.4) | −2.1 (−3.2 to −1.0) | −1.6 (−2.6 to −0.7) | 0.262 | <0.001 | <0.001 |
| Lung total elastic power (J/min) | 2.3 (1.2 to 3.6) | −0.3 (−1.5 to 0.4) | −2.5 (−3.9 to −1.6) | <0.001 | 0.188 | <0.001 |
| Lung total elastic power normalized to EELV (J/min/L) | 0.1 (−0.2 to 0.7) | −1.4 (−2.3 to −0.6) | −1.3 (−2.2 to −0.8) | 0.538 | <0.001 | <0.001 |
| Lung elastic static power (J/min) | 2.7 (1.6 to 3.9) | −0.1 (−0.8 to 0.8) | −2.2 (−3.8 to −1.6) | <0.001 | 0.909 | <0.001 |
| Lung elastic static power normalized to EELV (J/min/L) | 0.4 (0.0 to 1.2) | −0.9 (−1.2 to −0.3) | −1.4 (−2.2 to −0.9) | 0.006 | <0.001 | <0.001 |
| Lung elastic dynamic power (J/min) | 0.0 (−0.5 to 0.0) | −0.5 (−0.7 to 0.0) | 0.0 (−0.4 to 0.0) | 0.029 | 0.002 | 0.150 |
| Lung elastic dynamic power normalized to EELV (J/min/L) | −0.4 (−0.8 to −0.2) | −0.6 (−0.9 to −0.4) | 0.0 (−0.2 to 0.1) | <0.001 | <0.001 | 0.163 |

Data are shown as median differences (95% CI). A ventilation strategy with PEEP based on the PEEP/FiO_2_ table was used as the baseline. For the comparison between supine and prone positioning, a ventilation strategy with esophageal pressure-guided PEEP was used. Pairwise comparisons were performed using the Wilcoxon matched-pairs test. Hodges-Lehmann estimate was used to compute the median differences and 95% CIs between groups. PBW, predicted body weight; PEEP, positive end-expiratory pressure; FiO_2_, fraction of inspired oxygen; P_eso_, esophageal pressure; P_TP_, transpulmonary pressure; E_L_/E_RS_, elastance ratio of the lung to the respiratory system; EELV, end-expiratory lung volume; MP, mechanical power.

**Table S3** Effects of supine and prone positioning on gas exchange and hemodynamic parameters

|  | **Difference (95% CI)** | | | ***P* values** | | |
| --- | --- | --- | --- | --- | --- | --- |
| **Parameters** | **Supine versus Baseline** | **Prone versus**  **Baseline** | **Prone versus**  **Supine** | **Supine versus Baseline** | **Prone versus**  **Baseline** | **Prone versus**  **Supine** |
| PaO_2_/FiO_2_ (mmHg) | 37 (23 to 50) | 91 (57 to 129) | 36 (17 to 82) | <0.001 | <0.001 | <0.001 |
| Shunt fraction (%) | −10 (−13 to −7) | −16 (−21 to −11) | −2 (−7 to −1) | <0.001 | <0.001 | 0.009 |
| PaCO_2_ (mmHg) | 0.0 (−0.8 to 0.9) | 0.4 (−1.5 to 1.9) | −0.5 (−1.4 to 1.7) | 0.528 | 0.592 | 0.955 |
| pHa | 0.0 (0.0 to 0.0) | 0.0 (0.0 to 0.0) | 0.0 (0.0 to 0.0) | 0.709 | 0.715 | 0.335 |
| Alveolar dead space fraction | 0.0 (−0.01 to 0.01) | −0.03 (−0.06 to 0.0) | −0.03 (−0.04 to 0.0) | 0.989 | 0.003 | 0.001 |
| Heart rate (bpm) | 0 (0 to 1) | 2 (−2 to 5) | 1 (−2 to 4) | 0.291 | 0.393 | 0.448 |
| Mean arterial pressure (mmHg) | −1 (−4 to 1) | 6 (2 to 9) | 8 (2 to 11) | 0.024 | 0.003 | <0.001 |
| Central venous pressure (mmHg) | 2 (1 to 3) | 3 (1 to 4) | 1 (0 to 3) | <0.001 | 0.001 | 0.207 |
| Norepinephrine (μg/kg/min) | 0.0 (0.0 to 0.0) | 0.0 (0.0 to 0.0) | 0.0 (0.0 to 0.0) | 0.417 | 0.196 | 0.133 |
| Cardiac output (L/min) | −0.5 (−0.9 to −0.1) | 0.1 (−0.2 to 0.5) | 0.6 (0.3 to 1.0) | <0.001 | 0.809 | <0.001 |
| Oxygen delivery (mL/min) | 0 (−60 to 30) | 70 (1 to 141) | 97 (12 to 156) | 0.133 | <0.001 | <0.001 |

Data are shown as median differences (95% CI). A ventilation strategy with PEEP based on the PEEP/FiO_2_ table was used as the baseline. For the comparison between supine and prone positioning, a ventilation strategy with esophageal pressure-guided PEEP was used. During the study period, norepinephrine was required in 48 patients. Pairwise comparisons were performed using the Wilcoxon matched-pairs test. Hodges-Lehmann estimate was used to compute the median differences and 95% CIs between groups. PEEP, positive end-expiratory pressure; FiO_2_, fraction of inspired oxygen; P_eso_, esophageal pressure; PaO_2_/FiO_2_, arterial partial pressure of oxygen divided by fraction of inspired oxygen; PaCO_2_, arterial partial pressure of carbon dioxide; pHa, negative logarithm of the molar concentration of dissolved hydronium ions in arterial blood.

# Abbreviations

ΔP_RS_ respiratory driving pressure

ΔP_TP_ transpulmonary driving pressure

EELV end-expiratory lung volume

E_L_ static lung elastance

E_RS_ static respiratory system elastance

E_L_/E_RS_ elastance ratio of the lung to the respiratory system

FiO_2_ fraction of inspired oxygen

PaCO_2_ arterial partial pressure of carbon dioxide

PEEP positive end-expiratory pressure

P_peakRS_ peak airway pressure

P_eso_ esophageal pressure

P_platRS_ airway plateau pressure

P_TP_ transpulmonary pressure

V_T_ tidal volume

References

1. Brower RG, Lanken PN, MacIntyre N, Matthay MA, Morris A, Ancukiewicz M, Schoenfeld D, Thompson BT, National Heart Lung Blood Institute, ARDS Clinical Trials Network: Higher versus lower positive end-expiratory pressures in patients with the acute respiratory distress syndrome. N Engl J Med 2004, 351(4):327-336.

2. Nishida T, Suchodolski K, Schettino GP, Sedeek K, Takeuch M, Kacmarek RM: Peak volume history and peak pressure-volume curve pressures independently affect the shape of the pressure-volume curve of the respiratory system. Crit Care Med 2004, 32(6):1358-1364.
